# Genes associated with the *cis*-regulatory functions of intragenic LINE-1 elements

**DOI:** 10.1186/1471-2164-14-205

**Published:** 2013-03-27

**Authors:** Wachiraporn Wanichnopparat, Kulachanya Suwanwongse, Piyapat Pin-on, Chatchawit Aporntewan, Apiwat Mutirangura

**Affiliations:** 1 Center of Excellence in Molecular Genetics of Cancer and Human Diseases, Department of Anatomy, Faculty of Medicine, Chulalongkorn University, Bangkok, 10330, Thailand; 2 Inter-Department Program of Biomedical Sciences, Faculty of Graduate School, Chulalongkorn University, Bangkok, 10330, Thailand; 3 Department of Mathematics and Computer Science, Faculty of Science, Chulalongkorn University, Rama IV, Bangkok, 10330, Thailand

**Keywords:** Long interspersed element-1, *cis*-regulatory function, Expression profiling arrays, Intragenic LINE-1s, Gene body regulation, Regulatory network, LINE-1 methylation

## Abstract

**Background:**

Thousands of intragenic long interspersed element 1 sequences (LINE-1 elements or L1s) reside within genes. These intragenic L1 sequences are conserved and regulate the expression of their host genes. When L1 methylation is decreased, either through chemical induction or in cancer, the intragenic L1 transcription is increased. The resulting L1 mRNAs form RISC complexes with pre-mRNA to degrade the complementary mRNA. In this study, we screened for genes that are involved in intragenic L1 regulation networks.

**Results:**

Genes containing L1s were obtained from L1Base (http://l1base.molgen.mpg.de). The expression profiles of 205 genes in 516 gene knockdown experiments were obtained from the Gene Expression Omnibus (GEO) (http://www.ncbi.nlm.nih.gov/geo). The expression levels of the genes with and without L1s were compared using Pearson’s chi-squared test. After a permutation based statistical analysis and a multiple hypothesis testing, 73 genes were found to induce significant regulatory changes (upregulation and/or downregulation) in genes with L1s. In detail, 5 genes were found to induce both the upregulation and downregulation of genes with L1s, whereas 27 and 37 genes induced the downregulation and upregulation, respectively, of genes with L1s. These regulations sometimes differed depending on the cell type and the orientation of the intragenic L1s. Moreover, the siRNA-regulating genes containing L1s possess a variety of molecular functions, are responsible for many cellular phenotypes and are associated with a number of diseases.

**Conclusions:**

Cells use intragenic L1s as *cis*-regulatory elements within gene bodies to modulate gene expression. There may be several mechanisms by which L1s mediate gene expression. Intragenic L1s may be involved in the regulation of several biological processes, including DNA damage and repair, inflammation, immune function, embryogenesis, cell differentiation, cellular response to external stimuli and hormonal responses. Furthermore, in addition to cancer, intragenic L1s may alter gene expression in a variety of diseases and abnormalities.

## Background

Long interspersed element-1 sequences (LINE-1 elements or L1s) are broadly distributed throughout the human genome and are thought to have no physiological function in cells [1]. However, we recently demonstrated that intragenic L1s, which are L1s located inside a gene body, particularly in an intron, may regulate gene expression [2]. First, compared with intergenic L1s, human intragenic L1s contain conserved CpG dinucleotides at the 5’ UTR and sequences that are important for L1 transcription. Second, genes containing L1s are frequently downregulated in cancer and hypomethylated normal cells. Third, genes containing L1s are upregulated in argonaute 2 (AGO2)-downregulated embryonic kidney cell lines. L1 hypomethylation, both in cancer and as a result of chemical induction, increases the quantity of intragenic L1RNAs. The L1RNA forms a complex with its host gene pre-mRNA and AGO2. Consequently, the mRNA levels of genes containing L1s are repressed in cancer [2]. In this study, we explored whether the knockdown of other genes, in addition to AGO2, alters the mRNA level of genes with L1s. The resulting information should lead to interesting hypotheses of how intragenic L1s regulate gene expression and might indicate the biological processes in which this regulation is involved.

There are more than 500,000 copies of L1s in the human genome [3]. Most L1 elements are truncated at either the 5’ region or the 3’ region [4]. The full length of a putatively active L1 is approximately 6,000 base pairs, including a 5’ untranslated region (5’UTR) containing promoters, open reading frame 1 (ORF1), open reading frame 2 (ORF2) and a polyadenylation site in the 3’ untranslated region (3’UTR) [5]. In this study, we analyzed more than 10,000 L1s from the L1Base database (http://line1.bioapps.biozentrum.uni-wuerzburg.de/l1base.php) [4] that are longer than 4.5 kb and contain a 5’ UTR. More than 2,000 of these L1s are intragenic and reside within more than 1,000 genes.

Although L1s are considered “junk” DNA [6], there are several lines of evidence that support a role for intragenic L1s as *cis*-regulatory elements that play a crucial role in cell differentiation and the maintenance of normal cellular function. Lower L1 methylation levels are associated with reduced expression levels of the genes containing these L1s [2]. The methylation levels of intragenic L1s are tissue specific [7]. Consequently, one of the mechanisms that lead to different gene expression levels in different tissues may be a consequence of the epigenetic modification of intragenic L1s [8,9].

There are several mechanisms by which L1s may regulate gene expression. Most of the known L1-related gene regulatory mechanisms are mediated by L1RNA. One of these mechanisms is involved in X-inactivation; during this process, L1 mRNA forms facultative heterochromatin in the inactivated region [10-12]. An antisense 5’ L1 promoter can transcribe RNA from antisense DNA sequences at the 5’ end of L1 [13-16]. Moreover, the transcription from L1 can extend beyond the L1 poly-A sequence and produce RNA from unique DNA sequences that exist beyond the 3’ end of L1 [13,17]. In addition, we proved the intragenic L1s regulate the expression of genes containing these L1s [2]. Furthermore, there are other mechanisms by which interspersed repetitive sequences can regulate genes. For example, *Alu* can mediate alternative splicing, and the LTR of human endogenous retrovirus has been reported to possess enhancer function [18-21].

Recently, we compared the regulated mRNA levels of genes with L1s and those of genes without L1s through Pearson’s chi-squared analyses using an expression microarray of AGO2-knockdown cells [2]. We found that genes containing L1s were significantly upregulated in *AGO2*-knockdown cells. These data prompted us to further explore whether AGO2 plays a role in the control of gene expression through intragenic L1s [2].

In this study, we used publicly available data obtained from online sources to screen for genes that interact with intragenic L1s to control gene expression. In particular, we extracted the expression profiles obtained by gene knockdown experiments from the Gene Expression Omnibus repository (GEO datasets: http://www.ncbi.nlm.nih.gov/gds) [22,23] and acquired information regarding intragenic L1s from the L1Base database (http://line1.bioapps.biozentrum.uni-wuerzburg.de/l1base.php) [4]. The Connection Up- and Down-Regulation Expression Analysis of Microarrays (CU-DREAM) software package (http://pioneer.netserv.chula.ac.th/~achatcha/cu-dream/) [24] was used to perform various statistical analyses, including Student’s t-test and Pearson’s chi-squared test, to analyze the gene regulatory functions of intragenic L1s. We found that many genes regulate the genome-wide mRNA expression through regulatory networks of intragenic L1s. Therefore, intragenic L1s may serve as *cis*-regulatory elements that mediate gene expression in a variety of normal biological conditions and diseases.

## Methods

### Data collection and template preparation

Using “siRNA”, “shRNA” and “gene knockdown” as keywords, the expression profiles from microarray experiments that are related to the topics of interest and were performed between March 2005 and October 2011 were obtained (Additional file 1: Table S1). All of the supplementary information, including series matrix files and related platforms, which was freely available from the Gene Expression Omnibus repository (GEO datasets: http://www.ncbi.nlm.nih.gov/gds) [22,23], was downloaded. Subsequently, all of the GEO sample numbers (GSMs) were extracted for template preparation. In the template preparation process, the “control” samples included the samples that were labeled “scramble shRNA”, “mock experiment” and “shRNA or siRNA of reporter gene”, whereas the samples that were labeled “shRNA or siRNA of gene” were defined as “experimental”. Moreover, the threshold parameter was set to a significance level of 0.01 for each regulation.

### L1 library preparation

The putative L1 elements in the human genome were collected from L1Base (http://line1.bioapps.biozentrum.uni-wuerzburg.de/l1base.php) [4]. Only intragenic L1s were selected, and their host genes were compiled in a library, as described in a previous study [2]. In this study, 3 categories of intragenic L1 were used to build the library. First, the genes containing all types of intragenic L1s formed “Intragenic L1 Library”. Second, the genes containing the sense strand of intragenic L1s were included in “Sense L1 Library”. Third, the “Antisense L1 Library” included those genes that contained the antisense strand of intragenic L1s. These libraries were primarily used for the extension programming of the CU-DREAM software package.

### Statistical analysis

**Figure 1:**
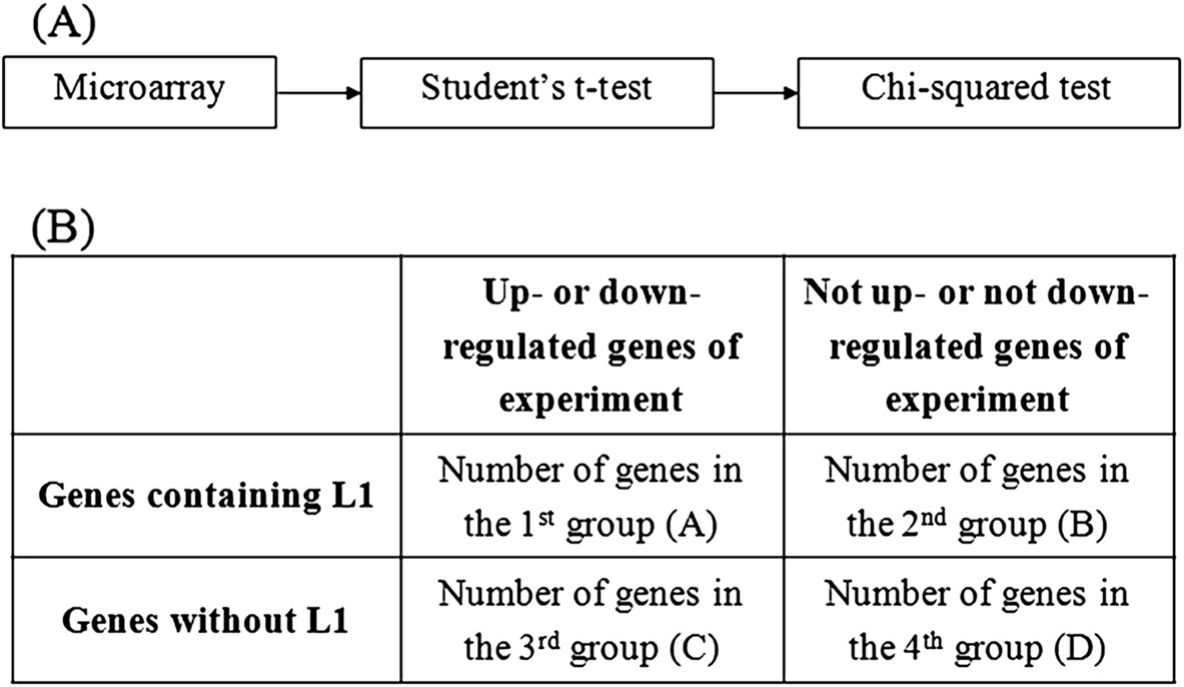
**The CU-DREAM extension program. (A)** A flow chart illustrating how the microarray data were processed. First, the microarray data for the fields of interest were collected and prepared. The program then computed the status of each gene (upregulated or downregulated) using Student’s t-test. Subsequently, the assessed genes were compared with a list of genes containing intragenic L1s. The results showed the associations between the gene regulation status and the presence of an L1 sequence in terms of ORs and p-values. (**B**) A table indicating the intersection between each experimental result and the genes containing intragenic L1s. These intersections are referred to as groups A through D. Group A includes the genes that are upregulated or downregulated and contain intragenic L1s. Group B includes the genes that are not upregulated or downregulated but contain intragenic L1s. Group C includes the genes that are upregulated or downregulated but do not contain intragenic L1s. Group D includes the remaining genes, which are not upregulated or downregulated and do not contain intragenic L1s.

First, the mRNA levels of the experimental and control samples were evaluated. Using the prepared templates of the microarrays, series matrix files and platforms, Student’s t-test was performed for each gene to compare the means of the control and experimental groups of the examined experiments. Each gene was then determined to be downregulated or upregulated based on the obtained p-value. Subsequently, the distributions of upregulated and downregulated genes were evaluated using Pearson’s chi-squared test to determine whether the distributions were dependent on the presence of an intragenic L1. Genes were classified into four groups, A through D. The significant genes with intragenic L1s that were downregulated or upregulated were included in group A. The significant genes without intragenic L1s were included in group B, whereas the non-significant genes with intragenic L1s were included in group C. The remaining genes (non-significant genes without L1s) were included in group D. The values of odds ratio (OR), p-values, and lower and upper 95% confidence interval (CI) of the genes in groups A through D were displayed in an MS Excel format. All of the statistical analyses were performed using extensions in the CU-DREAM software (http://pioneer.netserv.chula.ac.th/~achatcha/cu-dream/) [24] (Figure 1).

We also provide the p-values from a permutation test. Each gene was labeled “down,” “up”, or “neutral.” We randomly permuted or shuffled these labels to produce 100,000 replicates. Multiple hypothesis testing was corrected through false discovery rate (FDR) analysis [25]. The R Statistics software with the QVALUE package was used with the default parameters, with the exception that “Bootstrap” was used instead of “Smoother” [26]. We performed the FDR analysis on 516 siRNA experiments × {Down, Up} × {L1, sense L1, antisense L1} to obtain 3,096 permutation p-values. With the restriction of the q-value to less than 0.05, the number of significant *p*-values was found to be 230. We also obtained ${\hat{\pi }}_{0}=0.8293$, which is consistent with the low number of significant *p*-values.

### Data analysis

The results of the correlation of the gene knockdown experiments and the L1 libraries included the number of genes in groups A through D, as well as the ORs, 95% CIs, p-values, permuted p-values and q-values, which were organized by the direction of regulation. Next, the assessed experiments were grouped according to the OR value based on the direction of regulation. Thus, this analysis yielded 7 groups: the downregulation of genes with L1s with an OR greater than 1 (and the upregulation of these genes with an OR less than 1); the upregulation of genes with L1s with an OR greater than 1 (and the downregulation of these genes with an OR less than 1); the downregulation of genes with L1s with an OR less than 1; the upregulation of genes with L1s with an OR less than 1; both the downregulation and the upregulation of genes with L1s with an OR greater than 1; and both the downregulation and the upregulation of genes with L1s with an OR less than 1. The genes that could not be classified using these criteria were placed into a group of non-significant results. The official names of the identified genes and their functions were evaluated.

## Results

In this study, we screened hundreds of expression microarray experiments that were reported in the GEO and involved genes that were knocked down using siRNA or shRNA. The intragenic L1 elements, which were collected from L1base, contained 5’UTRs and at least 4,500 base pairs. The genes containing these intragenic L1 elements were extracted to build a library of 1,454 genes. All of the experiments were analyzed by Pearson’s chi-square test, multiple hypothesis testing and permutation based statistical analysis. The results were divided into 7 groups based on the direction of regulation (up or down) and the OR values. In this manuscript, we report the results of the analyses of 516 experiments, which represent 205 individual gene knockdowns (Table 1).

**Table 1:** A summary of the assessed gene knockdown experiments

**Direction of expression changes for genes with intragenic L1s and magnitude of the OR value**	**No. of experiments**	**No. of genes**	**Repeats/co-transfections**	**miRNA experiments**
Down and Up < 1	0	0	0	0
Down and Up > 1	7	4	1	2
Down < 1	9	10	1	0
Down > 1	40	26	13	2
Up < 1	12	11	3	0
Up > 1	39	32	8	4
non-significant	409	172	241	13
**Total**	**516**	**255**	**267**	**21**

Two examples of Pearson’s chi-squared tests are shown in two 2 × 2 tables (Tables 2 and 3). The first example shows a siRNA that promoted the downregulation of genes containing intragenic L1s, whereas the second shows a siRNA that promoted the upregulation of these genes. The genes that were downregulated in the *XIAP* knockdown experiments were compared with the list of genes containing intragenic L1s. Twenty-eight genes containing intragenic L1 elements were also downregulated in the *XIAP* knockdown experiments. In contrast, 1,312 genes with intragenic L1 elements were not downregulated in the *XIAP* knockdown experiments. Moreover, 169 genes did not contain intragenic L1s but were nevertheless downregulated by *XIAP* knockdown. The remaining 18,865 genes were not significantly affected by *XIAP* knockdown and did not contain intragenic L1s. The OR was 2.38, and the p-value was 1.39 × 10^-5^. The permuted p-value was 8.00 × 10^-5^, and the q-value was 2.12 × 10^-3^. This result implies a role for *XIAP* in the upregulation of the expression of genes containing L1s (Table 2).

**Table 2:** **A 2 × 2 table indicating the results of the *XIAP *siRNA experiment, which exhibited an OR in the downregulation direction**

***XIAP*siRNA**	**Downregulated**	**Not downregulated**	**OR = 2.38 P-value = 1.39 × 10**^**-5**^
**L1**	28	1,312	
**No L1**	169	18,865	
**Permuted p-value = 8.00 × 10**^**-5**^**, q-value = 2.12 × 10**^**-3**^			

**Table 3:** **A 2 × 2 table indicating the results of an *IKBKAP *siRNA experiment, which exhibited an OR in the upregulation direction**

***IKBKAP *siRNA**	**Upregulated**	**Not upregulated**	**OR = 2.22 P-value = 3.90 × 10**^**-4**^
**L1**	22	1,315	
**No L1**	143	18,976	
**Permuted p-value = 9.30 × 10**^**-4**^**, q-value = 1.56 × 10**^**-2**^			

The genes that were upregulated in the *IKBKAP* knockdown experiments were intersected with genes containing intragenic L1s. A total of 22 genes that contained intragenic L1s were upregulated in the *IKBKAP* knockdown experiments, whereas 143 genes that were upregulated in the *IKBKAP* knockdown experiments did not contain intragenic L1s. In addition, 1,315 genes contained intragenic L1s but were not upregulated in the *IKBKAP* knockdown experiments, and 18,976 genes were not upregulated in the *IKBKAP* knockdown experiments and did not contain intragenic L1s. The OR of this association was 2.22, and the p-value was 3.90 × 10^-4^. The permuted p-value and the q-value were 9.30 × 10^-4^ and 1.56 × 10^-2^, respectively (Table 3). Therefore, *IKBKAP* represses many genes with intragenic L1s.

**Figure 2:**
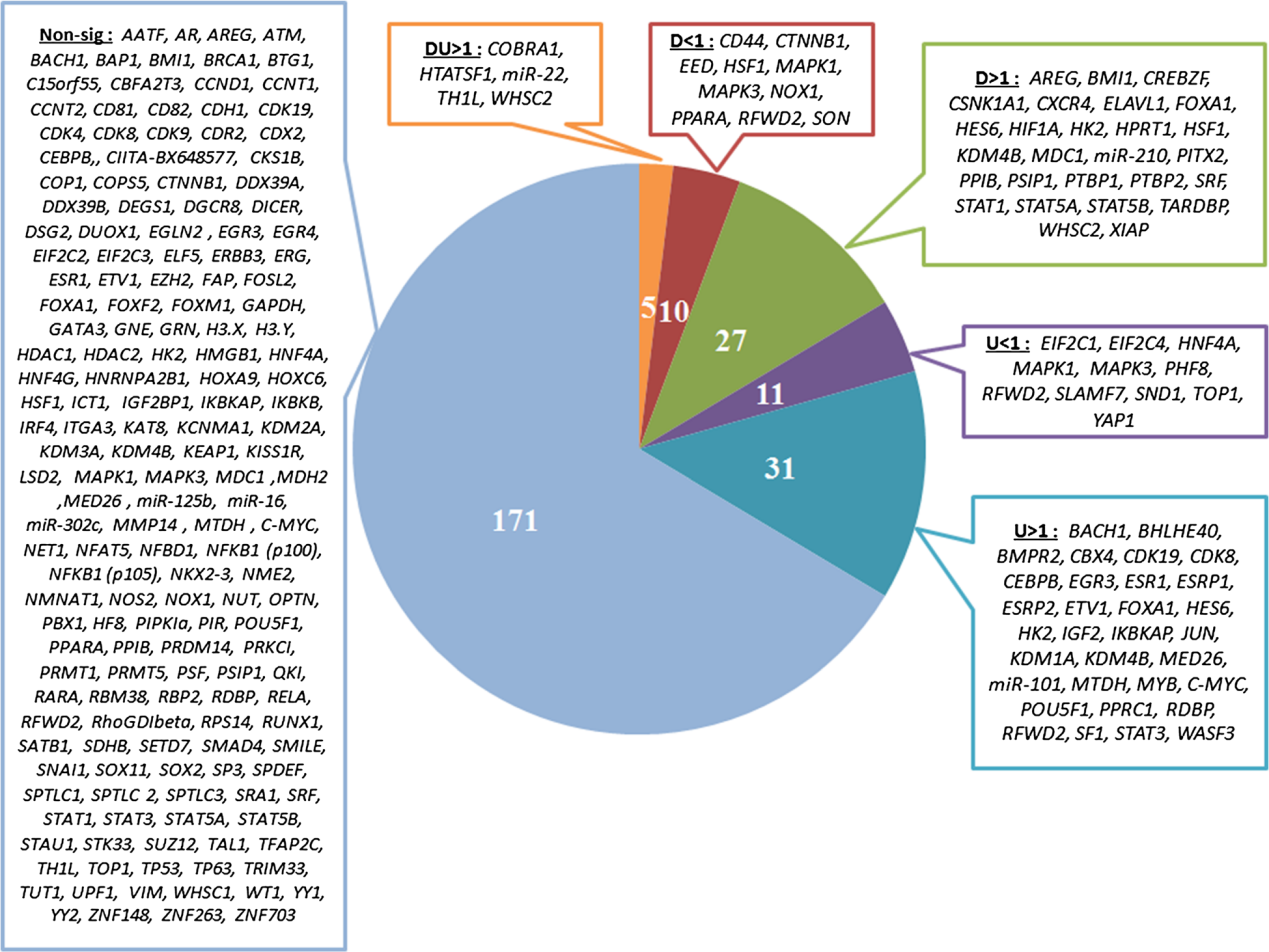
**The pie chart illustrates the number of genes and the official gene names for the siRNA experiments.** The results are grouped in terms of the direction of the expression change, the OR and the q-value.

The following results, which are shown in Table 1, Figure 2 and Additional file 1: Table S1, were obtained from the assessed experiments: no gene knockdowns were associated with the downregulation and upregulation of genes with L1s with an OR less than 1; 7 knockdowns were associated with the downregulation and upregulation of genes with L1s with an ORs greater than 1; 9 knockdowns were associated with the downregulation of genes with L1s with an OR less than 1; 40 knockdowns were associated with the downregulation of genes with L1s with an OR greater than 1 (and the upregulation of these genes with an OR less than 1); 12 knockdowns were associated with the upregulation of genes with L1s with an OR less than 1; and 39 knockdowns were associated with the upregulation of genes with L1s with an OR greater than 1 (and the downregulation of these genes with an OR less than 1). In the GSEs that exhibited the upregulation of genes with L1s with significant ORs (greater than 1), the OR values indicated increases in the mRNA levels of genes containing L1s. In the GSEs involving the downregulation of L1-containing genes with significant ORs (greater than 1), the OR values indicated decreases in the mRNA levels of genes containing L1s.

**Figure 3:**
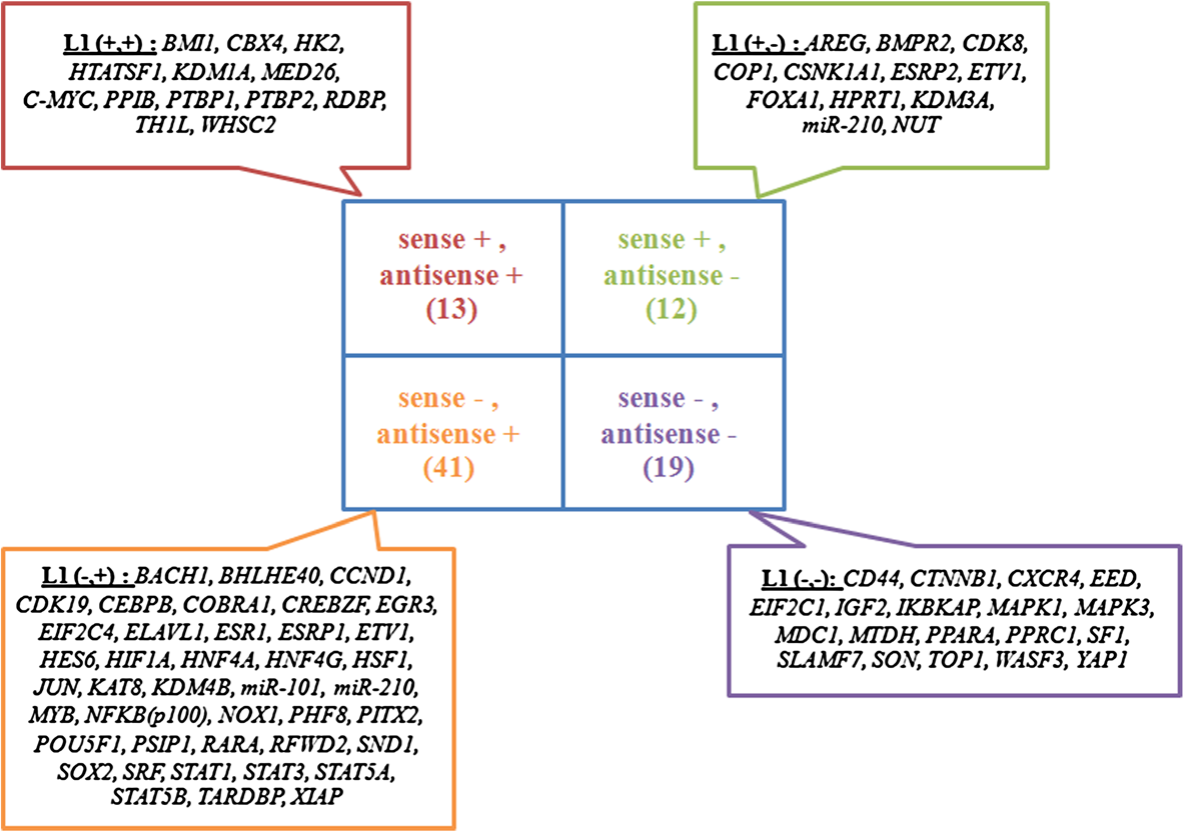
The diagram shows the 4 groups of significant siRNA genes that are associated with strand of L1s: (1) siRNA genes that were associated with genes containing sense L1s or antisense L1s (+, +), (2) siRNA genes that were associated with genes containing only sense L1s (+, -), (3) siRNA genes that were associated with genes containing only antisense L1s (−, +) and (4) siRNA genes that were not associated with genes containing sense L1s or antisense L1s (−, -) but were associated with genes containing both sense and antisense L1s.

Host genes of sense and antisense L1s were used to build the libraries and were analyzed by Pearson’s chi-square test and a permutation test using all of the experiments and all of the intragenic L1s. The results revealed that 15 and 12 experiments promoted the downregulation (OR > 1) and upregulation (OR > 1), respectively, of genes containing sense L1s. In contrast, 4 significant groups contained genes with antisense L1s: 39 experiments were associated with an OR greater than 1 with downregulation, 4 experiments were associated with an OR less than 1 with upregulation, 28 experiments were associated with an OR greater than 1 with upregulation, and 3 experiments were associated with an OR greater than 1 with downregulation and upregulation. Using strand-dependent intragenic L1s, the significant siRNA genes were categorized into 4 groups. The first group contained 13 siRNA genes that were associated with genes containing sense L1s and genes containing antisense L1s. There were 12 siRNA genes associated with genes containing sense L1s, whereas 41 siRNA genes were associated with genes containing antisense L1s. The last group, which consisted of 19 siRNA genes, exhibited significant association only when all genes containing L1s were used in the analysis (Figure 3 and Additional file 1: Table S1).

Data from more than one siRNA experiment were available for several genes. Among these replicates, 10 genes exhibited the same pattern of regulation, 39 genes were not significantly deregulated in one of the replicates, and 7 genes demonstrated opposing patterns of regulation. Notably, several factors differed between these various experiments, including the cell type, the oligomers used, and the treatments and treatment times (Table 4).

**Table 4:** Genes with more than one expression profile

**Regulation**	** Genes**	** Experimental differences**
non-significant and significant	*AREG, BACH1, BMI1, CDK19, CDK8, CEBPB, CTNNB1, CXCR4, EGR3, ESR1, ETV1, FOXA1, HK2, HNF4A, HSF1, IKBKAP, KDM4B, MAPK1, MAPK3, MDC1, MED26, MTDH, MYC, NOX1, PHF8, POU5F1, PPARA, PPIB, PSIP1, RARA, RDBP, RFWD2, SRF, STAT1, STAT3, STAT5A, STAT5B, TH1L, TOP1*	Differences in cell types, oligomer sets, cell passages, cell treatments and times of transfection
Different regulation direction	*HES6, HK2, , HSF1, MAPK1, MAPK3, RFWD2, WHSC2,*	Differences in cell types, oligomer sets, cell passages, platforms, cell treatments and times of transfection
Same regulation direction	*BACH1, BMI1, HES6, HK2, HNF4A, HTATSF1, KDM4B, PTBP1, PTBP2, XIAP*	Differences in cell types, oligomer sets, cell passages, platforms, cell treatments and times of transfection

**Figure 4:**
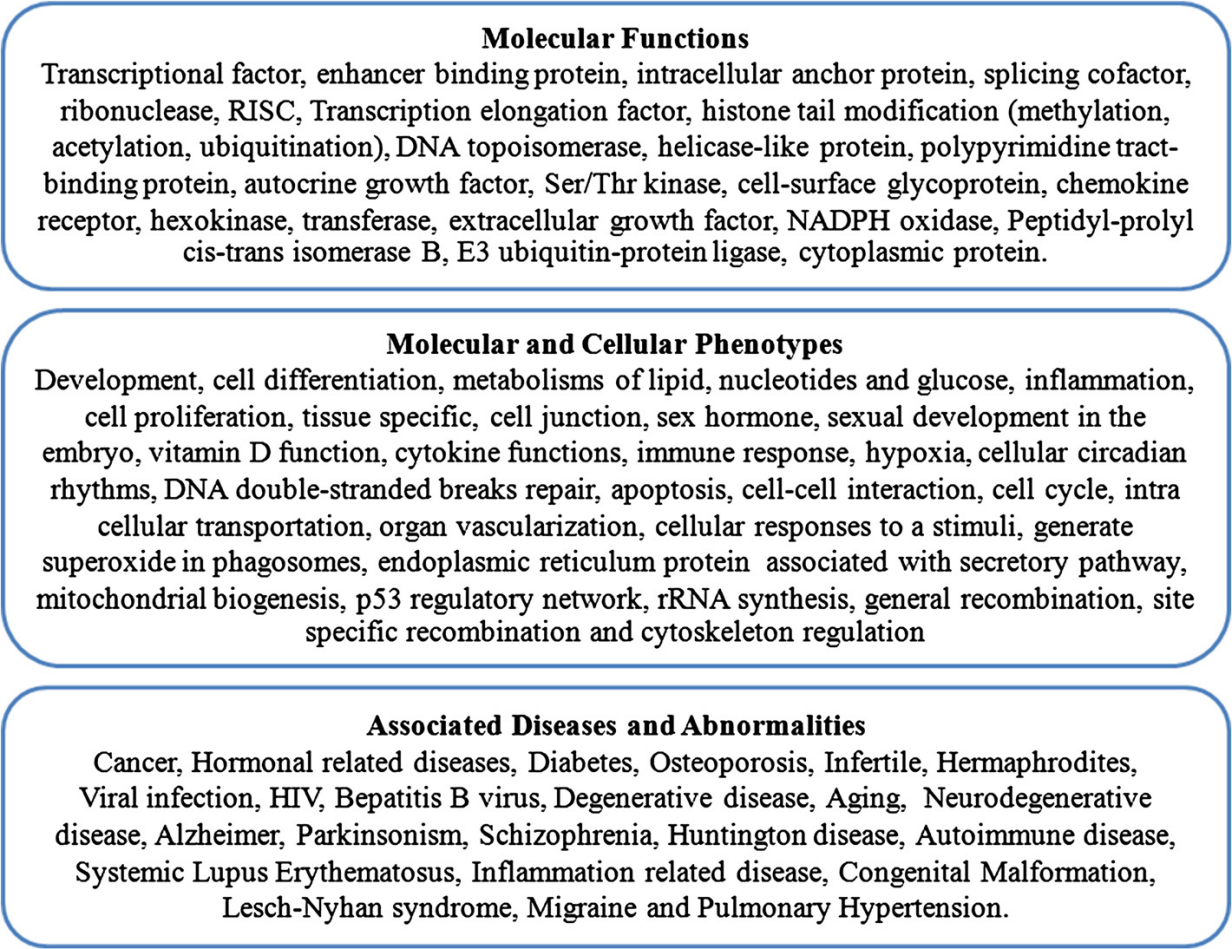
The figure shows the molecular functions, the molecular and cellular phenotypes and the associated diseases and abnormalities of significant siRNA genes.

We reviewed the genes that regulate genes with L1s, their molecular functions, and their association with cellular phenotypes. These genes produce several types of proteins, such as transcriptional factors, topoisomerase, histone modification, RNA elongation, signal transduction, membrane receptors and extracellular growth factors (Figure 4 and Additional file 2: Table S2). The genes regulating genes with L1s play a role in a number of cellular phenotypes, such as cell differentiation, cell proliferation, hormonal response, cell homeostasis, stem cell and viral infection, and have been reported to be associated with a number of diseases, such as cancer, hormone-related diseases, neurodegenerative diseases, schizophrenia, diabetes, and autoimmune and inflammation-related diseases (Figure 4 and Additional file 2: Table S2).

## Discussion

In this study, we evaluated hundreds number of expression profiles from gene knockdown experiments. Each Pearson’s chi-squared test compared the gene expression within the same array; consequently, the variations between different experiments did not interfere with each interpretation. In this study, we used FDR analysis [25] to correct the false positives by chance from multiple comparisons. We also performed permutation tests to exclude the possibility of obtaining a positive association with genes containing L1s by chance. Many siRNA treatments repress genes containing L1s. In contrast, several siRNA treatments increased the mRNA levels of genes with L1s. However, different results were obtained when the same genes were knocked down in different cell types, which indicated that several factors, some of which are tissue specific, are involved in intragenic L1-associated gene regulation mechanisms.

Numerous evidences support the hypothesis that there are several mechanisms by which intragenic L1s to serve as *cis*-regulatory elements. First, the orientation of intragenic L1s influences the intragenic L1-associated gene regulation for each siRNA experiment differently. Some genes regulate genes containing L1s only when the L1 orientations are sense or antisense only, whereas some genes demonstrated significant results regardless of the direction. Second, some siRNAs resulted in significant upregulation and downregulation. These results suggest that the intragenic L1 isoform changes or that some genes possess at least two different L1 regulation mechanisms: one mechanism promotes certain loci gene expression, and the other mechanism suppresses other genes with L1s. The 73 genes that were found to significantly regulate genes containing L1s possess a wide variety of functions. These genes produce transcriptional factors, enhancer binding proteins, topoisomerases, DNA double-strand break repair proteins, histone modification proteins, ribonucleases, RNA elongation factors, signal transduction proteins, membrane proteins and even extracellular proteins. Some of these genes may directly regulate genes containing L1s, which suggests multiple gene regulation mechanisms.

We recently reported the results of a Pearson’s chi-squared test that showed the role of AGO2 on the regulation of L1-containing genes. We confirmed the presence of the AGO2-pre mRNA-L1 RNA complex by immunoprecipitation assay [2]. In this study, we screened hundreds of genes to identify regulated L1-containing genes. Further experiments are required to confirm the effect and to define the mechanism by which individual gene knockdowns regulate the L1 expression.

Because changes in the expression of genes containing intragenic L1 sequences were found as a result of the knockdown of genes that affect a variety of cellular phenotypes or diseases, we hypothesize that the regulated L1-containing genes may be present in a wide range of biological processes, including diseases and abnormality. The 73 genes that were identified have the following functions: control of cell differentiation, cell proliferation, hormonal response, cell homeostasis, stem cell, immune response, genomic stability and viral infection. Furthermore, deregulations of these significant genes were associated with many diseases in addition to cancer, such as hormone-related diseases, neurodegenerative diseases, schizophrenia, diabetes, and autoimmune and inflammation-related diseases.

## Conclusions

Our study indicated an association between intragenic L1s and many genes that are mediators of genome-wide regulation. Therefore, L1s act as *cis*-regulatory elements. There are a number of genes that regulate genes with L1s. These regulatory genes possess a variety of molecular functions. This result suggests multiple regulatory mechanisms of gene control by intragenic L1s. Furthermore, based on the variable functions of the regulating genes, intragenic L1s may mediate several cellular phenotypes and are associated with the genome-wide gene expression observed in several diseases.

## Abbreviations

LINE-1 or L1: Long interspersed element-1; siRNA: Small interfering RNA; CU-DREAM: Connection Up- and Down-Regulation Expression Analysis of Microarrays program; FDR: False discovery rate; OR: Odds ratio; CI: Confidence interval; AGO2: Argonaute 2.

## Competing interests

The authors declare that they have no competing interests.

## Authors’ contributions

WW performed all experiments and analyzed data. KS and PP assisted performing experiments. CA conducted statistical analyses. AM conceived the idea for the project, designed all experiments and analyzed data. WW, CA and AM wrote the manuscript. All authors read and approved the final manuscript.

## Supplementary Material

Additional file 1: Table S1 Validated case and control samples and results from each experiment. Click here for file

Additional file 2: Table S2 Molecular functions and phenotypes of significant genes. Click here for file
