# Supplementary material for: Genes associated with the cis-regulatory functions of intragenic LINE-1 elements
Source: BMC Genomics. 2013 Mar 27;14:205. doi: 10.1186/1471-2164-14-205 (PMC3643820; doi:10.1186/1471-2164-14-205)
Supplement: Additional file 2: Table S2 — Molecular functions and phenotypes of significant genes. [file 1471-2164-14-205-S2.docx]

**Supplementary Table S2 Molecular functions and phenotypes of significant genes.**

| **Genes** | | **Functions** | |
| --- | --- | --- | --- |
|  |  | **Molecular functions** | **Phenotypes/Associated Diseases** |
| BHLHE40[[1](#_ENREF_1)] | | helix-loop-helix protein | Fatty acid oxidation in myocyte and a potential diabetic modifier gene |
| CEBPB[[2](#_ENREF_2)] | | interleukin 6-dependent CCAAT/enhancer binding protein | Immune and inflammatory response gene regulation |
| CTNNB1[[3](#_ENREF_3)] | | Intracellular anchor protein and transcriptional factor | Maintainance and regulate epithelial cell growth. Oncoprotein. |
| EGR3[[4](#_ENREF_4)] | | Zinc finger protein | Muscle, brain and lymphocyte function, associated with Schizophrenia [[5](#_ENREF_5)] |
| ESR1[[6](#_ENREF_6)] | | Estrogen nuclear receptor | Estrogen dependent gene transcription. Promote breast cancer and prevent osteoporosis |
| ETV1[[7](#_ENREF_7)] | | E twenty-six family of transcription factor | Proto-oncogene and Androgen receptor-regulated rene |
| FOXA1[[8](#_ENREF_8)] | | Forkhead class of DNA-binding protein | Estrogen receptor regulated gene. Tissue-specific gene expression. |
| HES6[[9](#_ENREF_9)] | | Helix-loop-helix | Promotes neuronal differentiation |
| HIF1A[[10](#_ENREF_10)] | | Alpha subunit of transcription factor hypoxia-inducible factor-1 (HIF-1) | Cellular response against hypoxia |
| HNF4A[[11](#_ENREF_11)] | | hepatocyte nuclear factor | Associated with maturity-onset diabetes of the young |
| HSF1[[12](#_ENREF_12)] | | Heat-shock transcription factor | Prevent DNA damage-associated senescence phenotype |
| HTATSF1[[13](#_ENREF_13)] | | General transcription factor | HIV-1 Tat cofactor for transcriptional elongation |
| JUN[[14](#_ENREF_14)] | | Transcription factor | Proto-oncogene |
| C-MYB[[15](#_ENREF_15)] | | Transcription factor | Hematopoietic differentiation |
| C-MYC[[16](#_ENREF_16)] | | Transcription factor | Proto-oncogene |
| PITX2[[17](#_ENREF_17)] | | Transcription factor | Cell proliferation and differentiation |
| POU5F1[[18](#_ENREF_18)] | | Transcription factor | Stem cell differentiation |
| PPARA[[19](#_ENREF_19)] | | Peroxisome proliferator-activated nuclear transcription  factor | Lipid metabolism. |
| PSIP1[[20](#_ENREF_20)] | | transcriptional co-activator and  pre-mRNA splicing coordinator | Cellular cofactor for viral DNA integration |
| SRF[[21](#_ENREF_21)] | | Transcription factor | Smooth muscle differentiation and proliferation |
| STAT1[[22](#_ENREF_22)] | | STAT protein family | Cytokine-mediated biological responses |
| STAT3[[22](#_ENREF_22)] | | STAT protein family | Cytokine-mediated biological responses |
| STAT5A[[22](#_ENREF_22)] | | STAT protein family | Cytokine-mediated biological responses |
| STAT5B[[22](#_ENREF_22)] | | STAT protein family | Cytokine-mediated biological responses |
| TARDBP[[23](#_ENREF_23)] | | DNA and RNA binding protein | Regulates transcription and splicing, affect HIV-1 replication, association with amyotrophic lateral sclerosis |
| YAP1[[24](#_ENREF_24)] | | Transcription coactivator, a protein binds to SH3 domain of YES protein | Oncoprotein |
| WHSC2[[25](#_ENREF_25)] | | RNA-binding protein | Negative elongation factor |
| TH1L[[25](#_ENREF_25)] | | RNA-binding protein | Negative elongation factor |
| COBRA1[[26](#_ENREF_26)] | | Nuclear protein | Negative elongation factor, Cofactor of BRCA1-( transcription, DNA double-stranded breaks repair,  and recombination.) |
| SON[[27](#_ENREF_27)] [[28](#_ENREF_28)] | | DNA and RNA-binding protein | mRNA splicing cofactor represses HBV transcription |
| RDBP[[25](#_ENREF_25)] | | RNA-binding protein | Negative elongation factor |
| RARA[[29](#_ENREF_29)] | | Retinoic acid receptor, alpha | development,  differentiation, apoptosis, granulopoeisis, and transcription of clock genes. |
| MED26 [[25](#_ENREF_25)] | | RNA-binding protein | Transcription elongation factor |
| IKBKAP [[30](#_ENREF_30)] | | RNA-binding protein | Transcription elongation factor, inhibitor of kappa light polypeptide gene enhancer in B-cells, kinase complex-associated protein, Familial dysautonomia |
| CREBZF [[31](#_ENREF_31)] | | transcription factor | positive regulator of p53 |
| BMI1[[32](#_ENREF_32)] | | Polycomb protein | Hematopoietic differentiation |
| KDM1A[[33](#_ENREF_33)] | | Histone demethylase | Self-renewal and differentiation |
| KDM4B[[34](#_ENREF_34)] | | Histone demethylase | - |
| PHF8[[35](#_ENREF_35)] | | Histone lysine demethylase | rRNA synthesis[[35](#_ENREF_35)], Cytoskeleton regulation[[36](#_ENREF_36)] |
| TOP1[[37](#_ENREF_37)] | | DNA topoisomerase | - |
| MDC1[[38](#_ENREF_38)] | | nuclear protein interacts with DNA double-strand break protein complex | Intra-S-phase DNA damage checkpoint |
| EED[[39](#_ENREF_39)] | | Multimeric protein complexes | Mediates repression of gene activity through histone deacetylation |
| CBX4[[40](#_ENREF_40)] | | E3 SUMO protein ligase, monoubiquitination of histone H2A 'Lys-119' | transcriptionally repression, exp HOX genes |
| BACH1[[41](#_ENREF_41)] | | Helicase-like Protein | Interacts with BRCA1 for DNA break repair |
| EIF2C1[[42](#_ENREF_42)] | | RNA-binding protein in RNA interference mechanism | - |
| SND1[[43](#_ENREF_43)] | | a component of RNA-induced silencing complex | Early stage colon carcinogenesis |
| EIF2C4[[42](#_ENREF_42)] | | RNA-binding protein in RNA interference mechanism | - |
| ELAVL1[[44](#_ENREF_44)] | | RNA-binding protein | - |
| ESRP1 [[45](#_ENREF_45), [46](#_ENREF_46)] | | Epithelial cell-type-specific splicing regulator | - |
| ESRP2[[45](#_ENREF_45)] | Epithelial cell-type-specific splicing regulator | | - |
| PTBP1[[47](#_ENREF_47)] | Polypyrimidine tract-binding protein | | Neuronal cell differentiation[[48](#_ENREF_48)] |
| PTBP2[[49](#_ENREF_49)] | Polypyrimidine tract-binding protein | | Neuronal cell differentiation |
| SF1[[50](#_ENREF_50)] | Nuclear pre-mRNA splicing factor, RNA-binding protein | | - |
| AREG[51](#_ENREF_51)] | | Autocrine growth factor[ | Prognostic factor in colorectal cancer |
| BMPR2[[52](#_ENREF_52)] | | Receptor of Ser/Thr kinase | Primary pulmonary hypertension [[52](#_ENREF_52)] |
| CD44[[54](#_ENREF_54)] | | Cell-surface glycoprotein | Cell–cell interactions |
| CDK19[[57](#_ENREF_57)] | | Ser/Thr protein kinase | Cell cycle control |
| CDK8[[57](#_ENREF_57)] | | Ser/Thr protein kinase | Cell cycle control |
| CSNK1A1[[60](#_ENREF_60)] | | Serine/threonine protein kinases | DNA repair,  intracellular transport, cell division |
| CXCR4[[63](#_ENREF_63)] | | CXC chemokine receptor | Organ vascularization |
| HK2[[64](#_ENREF_64)] | | Hexokinase 2 | Noninsulin-dependent diabetes mellitus |
| HPRT1[[65](#_ENREF_65)] | | Transferase | Generation of purine nucleotides, Lesch-Nyhan syndrome |
| IGF2[[66](#_ENREF_66)] | | Extracellular growth factor | Growth promoting hormone during [gestation](http://en.wikipedia.org/wiki/Gestation) |
| MAPK1[[67](#_ENREF_67)] | | Serine/threonine-specific protein kinase | Directing cellular responses to a stimuli |
| MAPK3[[67](#_ENREF_67)] | | Serine/threonine-specific protein kinase | Directing cellular responses to a stimuli |
| MTDH[[68](#_ENREF_68)] | | Interact with a variety of proteins | Oncoprotein and also implicated in diverse physiological and pathological processes, such as development, inflammation, neurodegeneration, migraine and Huntington disease |
| NOX1[[69](#_ENREF_69)] | | NADPH oxidase 1 | Generate superoxide in phagosomes |
| PPIB [[70](#_ENREF_70)] | | Peptidyl-prolyl cis-trans isomerase B | endoplasmic reticulum protein associated with secretory pathway |
| PPRC1[[71](#_ENREF_71)] | | Peroxisome proliferator-activated receptor gamma coactivator-related protein | mitochondrial biogenesis |
| RFWD2[[72](#_ENREF_72)] | | E3 ubiquitin-protein ligase | a critical negative regulator of p53 |
| SLAMF7[[73](#_ENREF_73)] | | Natural killer cell receptor | Immune response |
| WASF3[[74](#_ENREF_74)] | | actin reorganization | Platelet and immune cell development |
| XIAP[[75](#_ENREF_75)] | | Apoptotic suppressor proteins | Cell apoptosis |

1. Takeshita S, Suzuki T, Kitayama S, Moritani M, Inoue H, Itakura M: **Bhlhe40, a potential diabetic modifier gene on Dbm1 locus, negatively controls myocyte fatty acid oxidation**. *Genes Genet Syst* 2012, **87**(4):253-264.

2. Toda K, Akira S, Kishimoto T, Sasaki H, Hashimoto K, Yamamoto Y, Sagara Y, Shizuta Y: **Identification of a transcriptional regulatory factor for human aromatase cytochrome P450 gene expression as nuclear factor interleukin-6 (NF-IL6), a member of the CCAAT/enhancer-binding protein family**. *Eur J Biochem* 1995, **231**(2):292-299.

3. Clevers H, Nusse R: **Wnt/beta-catenin signaling and disease**. *Cell* 2012, **149**(6):1192-1205.

4. Patwardhan S, Gashler A, Siegel MG, Chang LC, Joseph LJ, Shows TB, Le Beau MM, Sukhatme VP: **EGR3, a novel member of the Egr family of genes encoding immediate-early transcription factors**. *Oncogene* 1991, **6**(6):917-928.

5. Yamada K, Gerber DJ, Iwayama Y, Ohnishi T, Ohba H, Toyota T, Aruga J, Minabe Y, Tonegawa S, Yoshikawa T: **Genetic analysis of the calcineurin pathway identifies members of the EGR gene family, specifically EGR3, as potential susceptibility candidates in schizophrenia**. *Proc Natl Acad Sci U S A* 2007, **104**(8):2815-2820.

6. Smith CL, O'Malley BW: **Evolving concepts of selective estrogen receptor action: from basic science to clinical applications**. *Trends Endocrinol Metab* 1999, **10**(8):299-300.

7. Cai C, Hsieh CL, Omwancha J, Zheng Z, Chen SY, Baert JL, Shemshedini L: **ETV1 is a novel androgen receptor-regulated gene that mediates prostate cancer cell invasion**. *Mol Endocrinol* 2007, **21**(8):1835-1846.

8. Carroll JS, Liu XS, Brodsky AS, Li W, Meyer CA, Szary AJ, Eeckhoute J, Shao W, Hestermann EV, Geistlinger TR *et al*: **Chromosome-wide mapping of estrogen receptor binding reveals long-range regulation requiring the forkhead protein FoxA1**. *Cell* 2005, **122**(1):33-43.

9. Bae S, Bessho Y, Hojo M, Kageyama R: **The bHLH gene Hes6, an inhibitor of Hes1, promotes neuronal differentiation**. *Development* 2000, **127**(13):2933-2943.

10. Zhou X, Tu J, Li Q, Kolosov VP, Perelman JM: **Hypoxia induces mucin expression and secretion in human bronchial epithelial cells**. *Transl Res* 2012, **160**(6):419-427.

11. Ellard S, Colclough K: **Mutations in the genes encoding the transcription factors hepatocyte nuclear factor 1 alpha (HNF1A) and 4 alpha (HNF4A) in maturity-onset diabetes of the young**. *Hum Mutat* 2006, **27**(9):854-869.

12. Kim G, Meriin AB, Gabai VL, Christians E, Benjamin I, Wilson A, Wolozin B, Sherman MY: **The heat shock transcription factor Hsf1 is downregulated in DNA damage-associated senescence, contributing to the maintenance of senescence phenotype**. *Aging Cell* 2012, **11**(4):617-627.

13. Zhou Q, Sharp PA: **Tat-SF1: cofactor for stimulation of transcriptional elongation by HIV-1 Tat**. *Science* 1996, **274**(5287):605-610.

14. Bohmann D, Bos TJ, Admon A, Nishimura T, Vogt PK, Tjian R: **Human proto-oncogene c-jun encodes a DNA binding protein with structural and functional properties of transcription factor AP-1**. *Science* 1987, **238**(4832):1386-1392.

15. Fahl SP, Crittenden RB, Allman D, Bender TP: **c-Myb is required for pro-B cell differentiation**. *J Immunol* 2009, **183**(9):5582-5592.

16. Dominguez-Sola D, Ying CY, Grandori C, Ruggiero L, Chen B, Li M, Galloway DA, Gu W, Gautier J, Dalla-Favera R: **Non-transcriptional control of DNA replication by c-Myc**. *Nature* 2007, **448**(7152):445-451.

17. Huang Y, Huang K, Boskovic G, Dementieva Y, Denvir J, Primerano DA, Zhu GZ: **Proteomic and genomic analysis of PITX2 interacting and regulating networks**. *FEBS Lett* 2009, **583**(4):638-642.

18. Zangrossi S, Marabese M, Broggini M, Giordano R, D'Erasmo M, Montelatici E, Intini D, Neri A, Pesce M, Rebulla P *et al*: **Oct-4 expression in adult human differentiated cells challenges its role as a pure stem cell marker**. *Stem Cells* 2007, **25**(7):1675-1680.

19. Costet P, Legendre C, More J, Edgar A, Galtier P, Pineau T: **Peroxisome proliferator-activated receptor alpha-isoform deficiency leads to progressive dyslipidemia with sexually dimorphic obesity and steatosis**. *J Biol Chem* 1998, **273**(45):29577-29585.

20. Marshall HM, Ronen K, Berry C, Llano M, Sutherland H, Saenz D, Bickmore W, Poeschla E, Bushman FD: **Role of PSIP1/LEDGF/p75 in lentiviral infectivity and integration targeting**. *PLoS One* 2007, **2**(12):e1340.

21. Wang Z, Wang DZ, Hockemeyer D, McAnally J, Nordheim A, Olson EN: **Myocardin and ternary complex factors compete for SRF to control smooth muscle gene expression**. *Nature* 2004, **428**(6979):185-189.

22. Takeda K, Akira S: **STAT family of transcription factors in cytokine-mediated biological responses**. *Cytokine Growth Factor Rev* 2000, **11**(3):199-207.

23. Kabashi E, Valdmanis PN, Dion P, Spiegelman D, McConkey BJ, Vande Velde C, Bouchard JP, Lacomblez L, Pochigaeva K, Salachas F *et al*: **TARDBP mutations in individuals with sporadic and familial amyotrophic lateral sclerosis**. *Nat Genet* 2008, **40**(5):572-574.

24. Camargo FD, Gokhale S, Johnnidis JB, Fu D, Bell GW, Jaenisch R, Brummelkamp TR: **YAP1 increases organ size and expands undifferentiated progenitor cells**. *Curr Biol* 2007, **17**(23):2054-2060.

25. Narita T, Yamaguchi Y, Yano K, Sugimoto S, Chanarat S, Wada T, Kim DK, Hasegawa J, Omori M, Inukai N *et al*: **Human transcription elongation factor NELF: identification of novel subunits and reconstitution of the functionally active complex**. *Mol Cell Biol* 2003, **23**(6):1863-1873.

26. Starita LM, Parvin JD: **The multiple nuclear functions of BRCA1: transcription, ubiquitination and DNA repair**. *Curr Opin Cell Biol* 2003, **15**(3):345-350.

27. Ahn EY, DeKelver RC, Lo MC, Nguyen TA, Matsuura S, Boyapati A, Pandit S, Fu XD, Zhang DE: **SON controls cell-cycle progression by coordinated regulation of RNA splicing**. *Mol Cell* 2011, **42**(2):185-198.

28. Sun CT, Lo WY, Wang IH, Lo YH, Shiou SR, Lai CK, Ting LP: **Transcription repression of human hepatitis B virus genes by negative regulatory element-binding protein/SON**. *J Biol Chem* 2001, **276**(26):24059-24067.

29. Mark M, Kastner P, Ghyselinck NB, Krezel W, Dupe V, Chambon P: **[Genetic control of the development by retinoic acid]**. *C R Seances Soc Biol Fil* 1997, **191**(1):77-90.

30. Close P, Hawkes N, Cornez I, Creppe C, Lambert CA, Rogister B, Siebenlist U, Merville MP, Slaugenhaupt SA, Bours V *et al*: **Transcription impairment and cell migration defects in elongator-depleted cells: implication for familial dysautonomia**. *Mol Cell* 2006, **22**(4):521-531.

31. Lopez-Mateo I, Villaronga MA, Llanos S, Belandia B: **The transcription factor CREBZF is a novel positive regulator of p53**. *Cell Cycle* 2012, **11**(20):3887-3895.

32. Ding X, Lin Q, Ensenat-Waser R, Rose-John S, Zenke M: **Polycomb group protein Bmi1 promotes hematopoietic cell development from embryonic stem cells**. *Stem Cells Dev* 2012, **21**(1):121-132.

33. Adamo A, Sese B, Boue S, Castano J, Paramonov I, Barrero MJ, Izpisua Belmonte JC: **LSD1 regulates the balance between self-renewal and differentiation in human embryonic stem cells**. *Nat Cell Biol* 2011, **13**(6):652-659.

34. Fodor BD, Kubicek S, Yonezawa M, O'Sullivan RJ, Sengupta R, Perez-Burgos L, Opravil S, Mechtler K, Schotta G, Jenuwein T: **Jmjd2b antagonizes H3K9 trimethylation at pericentric heterochromatin in mammalian cells**. *Genes Dev* 2006, **20**(12):1557-1562.

35. Zhu Z, Wang Y, Li X, Xu L, Wang X, Sun T, Dong X, Chen L, Mao H, Yu Y *et al*: **PHF8 is a histone H3K9me2 demethylase regulating rRNA synthesis**. *Cell Res* 2010, **20**(7):794-801.

36. Asensio-Juan E, Gallego C, Martinez-Balbas MA: **The histone demethylase PHF8 is essential for cytoskeleton dynamics**. *Nucleic Acids Res* 2012, **40**(19):9429-9440.

37. D'Arpa P, Machlin PS, Ratrie H, 3rd, Rothfield NF, Cleveland DW, Earnshaw WC: **cDNA cloning of human DNA topoisomerase I: catalytic activity of a 67.7-kDa carboxyl-terminal fragment**. *Proc Natl Acad Sci U S A* 1988, **85**(8):2543-2547.

38. Goldberg M, Stucki M, Falck J, D'Amours D, Rahman D, Pappin D, Bartek J, Jackson SP: **MDC1 is required for the intra-S-phase DNA damage checkpoint**. *Nature* 2003, **421**(6926):952-956.

39. Margueron R, Justin N, Ohno K, Sharpe ML, Son J, Drury WJ, 3rd, Voigt P, Martin SR, Taylor WR, De Marco V *et al*: **Role of the polycomb protein EED in the propagation of repressive histone marks**. *Nature* 2009, **461**(7265):762-767.

40. Kagey MH, Melhuish TA, Wotton D: **The polycomb protein Pc2 is a SUMO E3**. *Cell* 2003, **113**(1):127-137.

41. Cantor SB, Bell DW, Ganesan S, Kass EM, Drapkin R, Grossman S, Wahrer DC, Sgroi DC, Lane WS, Haber DA *et al*: **BACH1, a novel helicase-like protein, interacts directly with BRCA1 and contributes to its DNA repair function**. *Cell* 2001, **105**(1):149-160.

42. Hock J, Meister G: **The Argonaute protein family**. *Genome Biol* 2008, **9**(2):210.

43. Tsuchiya N, Ochiai M, Nakashima K, Ubagai T, Sugimura T, Nakagama H: **SND1, a component of RNA-induced silencing complex, is up-regulated in human colon cancers and implicated in early stage colon carcinogenesis**. *Cancer Res* 2007, **67**(19):9568-9576.

44. Uren PJ, Burns SC, Ruan J, Singh KK, Smith AD, Penalva LO: **Genomic analyses of the RNA-binding protein Hu antigen R (HuR) identify a complex network of target genes and novel characteristics of its binding sites**. *J Biol Chem* 2011, **286**(43):37063-37066.

45. Warzecha CC, Shen S, Xing Y, Carstens RP: **The epithelial splicing factors ESRP1 and ESRP2 positively and negatively regulate diverse types of alternative splicing events**. *RNA Biol* 2009, **6**(5):546-562.

46. Revil T, Jerome-Majewska LA: **During embryogenesis, Esrp1 expression is restricted to a subset of epithelial cells and is associated with splicing of a number of developmentally important genes**. *Dev Dyn* 2012.

47. Ghetti A, Pinol-Roma S, Michael WM, Morandi C, Dreyfuss G: **hnRNP I, the polypyrimidine tract-binding protein: distinct nuclear localization and association with hnRNAs**. *Nucleic Acids Res* 1992, **20**(14):3671-3678.

48. Makeyev EV, Zhang J, Carrasco MA, Maniatis T: **The MicroRNA miR-124 promotes neuronal differentiation by triggering brain-specific alternative pre-mRNA splicing**. *Mol Cell* 2007, **27**(3):435-448.

49. Rahman L, Bliskovski V, Kaye FJ, Zajac-Kaye M: **Evolutionary conservation of a 2-kb intronic sequence flanking a tissue-specific alternative exon in the PTBP2 gene**. *Genomics* 2004, **83**(1):76-84.

50. Corioni M, Antih N, Tanackovic G, Zavolan M, Kramer A: **Analysis of in situ pre-mRNA targets of human splicing factor SF1 reveals a function in alternative splicing**. *Nucleic Acids Res* 2011, **39**(5):1868-1879.

51. Ohchi T, Akagi Y, Kinugasa T, Kakuma T, Kawahara A, Sasatomi T, Gotanda Y, Yamaguchi K, Tanaka N, Ishibashi Y *et al*: **Amphiregulin is a prognostic factor in colorectal cancer**. *Anticancer Res* 2012, **32**(6):2315-2321.

52. Deng Z, Morse JH, Slager SL, Cuervo N, Moore KJ, Venetos G, Kalachikov S, Cayanis E, Fischer SG, Barst RJ *et al*: **Familial primary pulmonary hypertension (gene PPH1) is caused by mutations in the bone morphogenetic protein receptor-II gene**. *Am J Hum Genet* 2000, **67**(3):737-744.

53. Satijn DP, Olson DJ, van der Vlag J, Hamer KM, Lambrechts C, Masselink H, Gunster MJ, Sewalt RG, van Driel R, Otte AP: **Interference with the expression of a novel human polycomb protein, hPc2, results in cellular transformation and apoptosis**. *Mol Cell Biol* 1997, **17**(10):6076-6086.

54. Aruffo A, Stamenkovic I, Melnick M, Underhill CB, Seed B: **CD44 is the principal cell surface receptor for hyaluronate**. *Cell* 1990, **61**(7):1303-1313.

55. Jin L, Hope KJ, Zhai Q, Smadja-Joffe F, Dick JE: **Targeting of CD44 eradicates human acute myeloid leukemic stem cells**. *Nat Med* 2006, **12**(10):1167-1174.

56. Mukhopadhyay A, Kramer JM, Merkx G, Lugtenberg D, Smeets DF, Oortveld MA, Blokland EA, Agrawal J, Schenck A, van Bokhoven H *et al*: **CDK19 is disrupted in a female patient with bilateral congenital retinal folds, microcephaly and mild mental retardation**. *Hum Genet* 2010, **128**(3):281-291.

57. Fisher D, Krasinska L, Coudreuse D, Novak B: **Phosphorylation network dynamics in the control of cell cycle transitions**. *J Cell Sci* 2012, **125**(Pt 20):4703-4711.

58. Firestein R, Bass AJ, Kim SY, Dunn IF, Silver SJ, Guney I, Freed E, Ligon AH, Vena N, Ogino S *et al*: **CDK8 is a colorectal cancer oncogene that regulates beta-catenin activity**. *Nature* 2008, **455**(7212):547-551.

59. Lu R, Misra V: **Zhangfei: a second cellular protein interacts with herpes simplex virus accessory factor HCF in a manner similar to Luman and VP16**. *Nucleic Acids Res* 2000, **28**(12):2446-2454.

60. Dubois T, Kerai P, Zemlickova E, Howell S, Jackson TR, Venkateswarlu K, Cullen PJ, Theibert AB, Larose L, Roach PJ *et al*: **Casein kinase I associates with members of the centaurin-alpha family of phosphatidylinositol 3,4,5-trisphosphate-binding proteins**. *J Biol Chem* 2001, **276**(22):18757-18764.

61. Jia J, Tong C, Wang B, Luo L, Jiang J: **Hedgehog signalling activity of Smoothened requires phosphorylation by protein kinase A and casein kinase I**. *Nature* 2004, **432**(7020):1045-1050.

62. Elyada E, Pribluda A, Goldstein RE, Morgenstern Y, Brachya G, Cojocaru G, Snir-Alkalay I, Burstain I, Haffner-Krausz R, Jung S *et al*: **CKIalpha ablation highlights a critical role for p53 in invasiveness control**. *Nature* 2011, **470**(7334):409-413.

63. Tachibana K, Hirota S, Iizasa H, Yoshida H, Kawabata K, Kataoka Y, Kitamura Y, Matsushima K, Yoshida N, Nishikawa S *et al*: **The chemokine receptor CXCR4 is essential for vascularization of the gastrointestinal tract**. *Nature* 1998, **393**(6685):591-594.

64. Deeb SS, Malkki M, Laakso M: **Human hexokinase II: sequence and homology to other hexokinases**. *Biochem Biophys Res Commun* 1993, **197**(1):68-74.

65. Sculley DG, Dawson PA, Emmerson BT, Gordon RB: **A review of the molecular basis of hypoxanthine-guanine phosphoribosyltransferase (HPRT) deficiency**. *Hum Genet* 1992, **90**(3):195-207.

66. Roth RA: **Structure of the receptor for insulin-like growth factor II: the puzzle amplified**. *Science* 1988, **239**(4845):1269-1271.

67. Seger R, Krebs EG: **The MAPK signaling cascade**. *FASEB J* 1995, **9**(9):726-735.

68. Yoo BK, Emdad L, Lee SG, Su ZZ, Santhekadur P, Chen D, Gredler R, Fisher PB, Sarkar D: **Astrocyte elevated gene-1 (AEG-1): A multifunctional regulator of normal and abnormal physiology**. *Pharmacol Ther* 2011, **130**(1):1-8.

69. Cheng G, Lambeth JD: **NOXO1, regulation of lipid binding, localization, and activation of Nox1 by the Phox homology (PX) domain**. *J Biol Chem* 2004, **279**(6):4737-4742.

70. Price ER, Zydowsky LD, Jin MJ, Baker CH, McKeon FD, Walsh CT: **Human cyclophilin B: a second cyclophilin gene encodes a peptidyl-prolyl isomerase with a signal sequence**. *Proc Natl Acad Sci U S A* 1991, **88**(5):1903-1907.

71. Vercauteren K, Gleyzer N, Scarpulla RC: **Short hairpin RNA-mediated silencing of PRC (PGC-1-related coactivator) results in a severe respiratory chain deficiency associated with the proliferation of aberrant mitochondria**. *J Biol Chem* 2009, **284**(4):2307-2319.

72. Dornan D, Wertz I, Shimizu H, Arnott D, Frantz GD, Dowd P, O'Rourke K, Koeppen H, Dixit VM: **The ubiquitin ligase COP1 is a critical negative regulator of p53**. *Nature* 2004, **429**(6987):86-92.

73. Boles KS, Mathew PA: **Molecular cloning of CS1, a novel human natural killer cell receptor belonging to the CD2 subset of the immunoglobulin superfamily**. *Immunogenetics* 2001, **52**(3-4):302-307.

74. Sossey-Alaoui K, Li X, Ranalli TA, Cowell JK: **WAVE3-mediated cell migration and lamellipodia formation are regulated downstream of phosphatidylinositol 3-kinase**. *J Biol Chem* 2005, **280**(23):21748-21755.

75. Deveraux QL, Takahashi R, Salvesen GS, Reed JC: **X-linked IAP is a direct inhibitor of cell-death proteases**. *Nature* 1997, **388**(6639):300-304.
